# Poly(L-Lactic Acid) Composite with Surface-Modified Magnesium Hydroxide Nanoparticles by Biodegradable Oligomer for Augmented Mechanical and Biological Properties

**DOI:** 10.3390/ma14195869

**Published:** 2021-10-07

**Authors:** Seung-Woon Baek, Duck Hyun Song, Ho In Lee, Da-Seul Kim, Yun Heo, Jun Hyuk Kim, Chun Gwon Park, Dong Keun Han

**Affiliations:** 1 Department of Biomedical Science, CHA University, 335 Pangyo-ro, Bundang-gu, Seongnam-si 13488, Korea; baiksw830@g.skku.edu (S.-W.B.); mondh920@naver.com (D.H.S.); 016rhk@naver.com (H.I.L.); dptmf4011@cau.ac.kr (D.-S.K.); yheo@chauniv.ac.kr (Y.H.); junkimyo@gmail.com (J.H.K.); 2 Department of Biomedical Engineering, SKKU Institute for Convergence, Sungkyunkwan University (SKKU), 2066 Seobu-ro, Jangan-gu, Suwon-si 16419, Korea; chunpark@skku.edu; 3 Department of Intelligent Precision Healthcare Convergence, SKKU Institute for Convergence, Sungkyunkwan University (SKKU), 2066 Seobu-ro, Jangan-gu, Suwon-si 16419, Korea; 4 School of Integrative Engineering, Chung-Ang University, 84 Heukseok-ro, Dongjak-gu, Seoul 06974, Korea

**Keywords:** poly(L-lactic acid), magnesium hydroxide, oligo(D,L-lactic acid), oligo(lactide-co-caprolactone), biodegradable vascular scaffold

## Abstract

Poly(L-lactic acid) (PLLA) has attracted a great deal of attention for its use in biomedical materials such as biodegradable vascular scaffolds due to its high biocompatibility. However, its inherent brittleness and inflammatory responses by acidic by-products of PLLA limit its application in biomedical materials. Magnesium hydroxide (MH) has drawn attention as a potential additive since it has a neutralizing effect. Despite the advantages of MH, the MH can be easily agglomerated, resulting in poor dispersion in the polymer matrix. To overcome this problem, oligo-L-lactide-ε-caprolactone (OLCL) as a flexible character was grafted onto the surface of MH nanoparticles due to its acid-neutralizing effect and was added to the PLLA to obtain PLLA/MH composites. The pH neutralization effect of MH was maintained after surface modification. In an in vitro cell experiment, the PLLA/MH composites including OLCL-grafted MH exhibited lower platelet adhesion, cytotoxicity, and inflammatory responses better than those of the control group. Taken together, these results prove that PLLA/MH composites including OLCL-grafted MH show excellent augmented mechanical and biological properties. This technology can be applied to biomedical materials for vascular devices such as biodegradable vascular scaffolds.

## 1. Introduction

Biodegradable polymers like polyglycolide, poly(L-lactic acid) (PLLA), and polycaprolactone are widely used in materials for biomedical applications. Among the biodegradable polymers, PLLA has attracted a great deal of attention for its use in biomedical materials such as biodegradable vascular scaffold (BVS) due to its high biocompatibility. Generally, thermal processing such as extrusion and injection molding is utilized to produce implant devices using PLLA [1,2]. PLLA is weak to thermal degradation because of its vulnerable thermal stability, which obstructs its wide medical applications. Thermal degradation of PLLA occurs via inter- or intramolecular transesterification reactions and hydrolysis while thermal processing not only decreases the mechanical properties and molecular weight of the products but also increases the biodegradation rate. PLLA also has limited applications in biomedical engineering and science such as tissue regeneration and medical devices because it has some drawbacks such as lower hydrophilicity, slow degradation rate, brittleness, and acidic degradation products [2,3]. Due to its semi-crystalline character, many studies have described that the PLLA can be blended with biodegradable and ductile polymers such as thermoplastic polyamide elastomer (PAE) [1], polyglycolic acid (PGA) [4,5], poly(butylene succinate) (PBS), hydroxyalkanoates (PHAs) [6,7], acrylate copolymer [8], poly(butylene adipate-co-terephthalate) (PBAT) [9], hyperbranched polymers (HBPs) [10,11], and soybean oil derivatives [12,13] to improve brittleness. Among them, the PLLA blend with oligo-L-lactide-ε-caprolactone (OLCL) has an aliphatic chain, flexible character, and low melting temperature, which can improve the mechanical properties of PLLA [14]. Additionally, PLCL, with its great compliance matching and mechanical properties with natural blood vessels, has already been approved by the FDA for clinical applications [15,16,17,18,19]. The in vivo degraded acidic by-products of PLLA can contribute to a noninfectious inflammatory response in the body. Overcoming the inflammatory problem of PLLA is important in the application to medical devices.

One of the inorganic biomaterials, magnesium hydroxide [Mg(OH)_2_, MH], is currently being used for potential medical applications in different areas, which include pH-responsive nanocarriers and antacid agents [20,21,22,23]. MH composites play an important role in various regenerative medical devices and tissue engineering treatment strategies. In bone regeneration, PLLA/MH successfully improved bone regeneration with significantly suppressed inflammatory responses [24]. In spiral fusion, MH composite revealed improved degradation performance, neutralization effects, and hydrophilicity [25]. In kidney regeneration, pH was mitigated in the degradation. For in vivo tests, renal tissue formation and glomerulus number were higher, and inflammation and fibrosis were lower [26]. In stents, the MH composites neutralized the acidification of the near tissue by the degraded polymer and reduced restenosis, inflammation, and late thrombosis [27]. A previous study reported that we confirmed the antagonistic effect of MH particles against the influx of acidic degradation products of PLGA and indicated that it inhibited re-endothelial activation caused by the accumulation of acidic degradation products of PLGA in human coronary artery endothelial cells (HCAECs) [28,29]. Although MH has a number of advantages as an anti-inflammatory in polymer-based devices, MH as the hydrophilic particle is difficult to evenly disperse in polymers as the hydrophobic matrix. Besides, poor dispersion stability can disrupt the interaction between polymer chains and decrease the toughness of polymer devices. In a prior study, an attempt was made to disperse MH nanoparticles modified with oligo-D,L-lactic acid (ODLLA) by two different grafting methods in the PLLA matrix [30].

**Figure 1:**
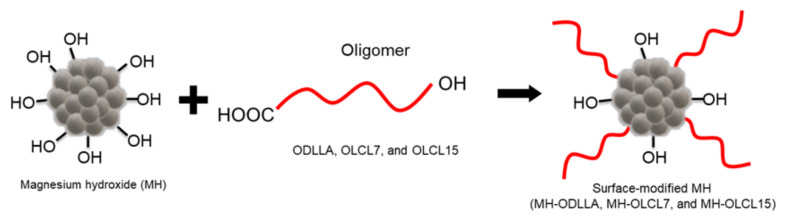
A schematic illustration for preparation of oligomer grafted MH nanoparticles.

In this study, we enhanced the dispersion stability of the MH in a hydrophobic polymer matrix by surface-grafting the MH with a hydrophobic and flexible oligomer to reduce inflammation through pH neutralization. The MH nanoparticles were grafted by ODLLA, low molecular weight of OLCL (OLCL7), and high molecular weight of OLCL (OLCL15) (Figure 1). In addition, PLLA/MH composites with OLCL-modified MH (MH-OLCL7 and MH-OLCL15) can improve brittleness, one of the most important disadvantages of PLLA. The physico-mechanical properties of the PLLA/MH composites were measured using a universal testing machine (UTM) and the MH content in the PLLA/MH composites was demonstrated through thermogravimetric analysis (TGA). We carried out degradation behavior to verify the pH neutralizing effect of the MH-OLCL-containing PLLA/MH composites. In addition, in vitro blood and biocompatibility experiments were accomplished to evaluate the blood–composite interactions as well as cytotoxicity and inflammatory effects.

The purpose of this study was to make and characterize a PLLA/MH composite with surface-modified magnesium hydroxide nanoparticles by biodegradable oligomers for augmented mechanical and biological properties to surmount previous problems of PLLA effectively, such as lower hydrophilicity, slow degradation rate, brittleness, and acidic degradation products.

## 2. Materials and Methods

### 2.1. Materials

PLLA (average Mw: 230 kDa) was provided by Total Corbion (Amsterdam, Netherlands). L-Lactide was purchased from DURECT Co. (Birmingham, AL, USA). DL-lactide was obtained from Tokyo Chemical Ind. (Tokyo, Japan). Magnesium hydroxide (MH, 99.5%), ε-caprolactone, fibrinogen from human plasma, stannous octoate, sodium dodecyl sulfate (SDS), L-(+)-lactic acid (L-Lac), and 1-octanol were obtained from Sigma Aldrich (Burlington, MA, USA). Platelet concentrates (5 × 10^4^ platelets per μL) were purchased from the Korean National Red Cross (Seoul, Korea). IL-6 and IL-8 enzyme-linked immunosorbent assay (ELISA) kits were obtained from R&D Systems (Minneapolis, MN, USA).

HCAECs were acquired from Cambrex (Walkersville, MD, USA) and EGM-2 media with an MV bullet kit (Lonza, Basel, Switzerland). A cell-counting kit (CCK-8) was obtained from Dongin LS (Seoul, Korea). Phosphate-buffered saline (PBS) solution was supported from Hyclone (GE Healthcare Life Sciences, Boston, MA, USA). A Micro BCA^TM^ Protein assay kit was acquired from Thermo Scientific TM (Waltham, MA, USA). All chemicals were laboratory reagent grade and used without purification.

### 2.2. Synthesis of Oligomers

ODLLA was synthesized by ring-opening polymerization as described elsewhere [30]. Briefly, D,L-lactide (10 mmol), 1-octanol (1 mmol), and stannous octoate (monomer/catalyst = 10,000) were added into a round flask with anhydrous toluene (100 mL). The flask was purged with a nitrogen atmosphere. The polymerization was conducted at 150 °C for 18 h.

OLCL was also synthesized by ring-opening polymerization such as ODLLA [31]. L-Lactide (157.399 mmol), ε-caprolactone (69.102 mmol), 1-octanol (0.77 or 3.84 mmol), and stannous octoate (monomer/catalyst = 5,000) were added into a round flask with anhydrous toluene (80 mL). The flask was purged with a nitrogen atmosphere. The polymerization was performed at 140 °C for 18 h.

All synthesized oligomers were dissolved in chloroform and precipitated into hexane then dried for 48 h.

### 2.3. Preparation of Oligomer-Grafted MH Nanoparticles

Fabrication of the oligomer-grafted MH was conjugated with hydroxylate of MH and carboxylate of the oligomer, as by grafting the previous study [30]. Consequently, the MH and oligomer were reacted under a vacuum at 150 °C for 15 h.

The mixture was dissolved in chloroform/acetone (4:6) and centrifuged for 10 min at 7000 rpm. The resultant products were dried for 24 h at room temperature.

### 2.4. Preparation of the PLLA/MH Composites

The fabrication process of the composite was completed in two steps. PLLA and oligomer-grafted MH were prepared as solvent-cast composites. PLLA (5 g) and oligomer-grafted MH (0.5 g) were dissolved in chloroform (70 mL). Chloroform was evaporated for 24 h at 25 °C. The resultant composites were dried for 48 h at 50 °C. The composites underwent a hot-pressing process using a compression molding machine (QM900A, QMESYS, Korea). The mold thickness was 250 μm and its dimensions were 10 × 10 mm^2^. The molds were heated to 180 °C for 5 min. After a melting time, the pressure increased and was retained for a press time of 2 min.

### 2.5. Analyses of Oligomers and Oligomer-Grafted MH

The molecular weight of the oligomers was established by gel permeation chromatography (GPC; HLC-8320GPC, TOSOH, Tokyo, Japan). The chemical bonding was established by attenuated total reflection-Fourier transform infrared (ATR-FTIR, PerkinElmer, Waltham, MA, USA) with a spectral resolution of 32 scans and 4 cm^−1^, at the scale of 400–4000 cm^−1^. The modifying degree and composition were determined by thermogravimetric analysis (TGA-4000, PerkinElmer, Waltham, MA, USA). The temperature range was from 25 to 800 °C, under a nitrogen atmosphere at a heating rate of 10 °C/min. The weight loss contrast temperature curve was measured. The morphology and size of nanoparticles were investigated by scanning electron microscopy (SEM, Hitachi S-4800, Ibaraki, Japan) and dynamic laser scattering (DLS, Malvern Panalytical, Malvern, UK).

### 2.6. Analyses of the PLLA/MH Composites

The morphology of the PLLA/MH composites was evaluated by field emission scanning electron microscopy (FE-SEM, Hitachi, Tokyo, Japan). The chemical composition of the sample was determined by energy dispersive spectroscopy (EDS). The grafting amount of PLLA/MH composite was measured by TGA. The infrared spectra of PLLA/MH composites were acquired by using ATR-FTIR.

The hydrophilicity of the surface PLLA combined with MH particles was examined by a contact angle analyzer (Phoenix 300, Surface Electro Optics, Suwon, Korea).

### 2.7. Mechanical Properties

Elongation, tensile strength, and Young’s modulus were determined by UTM (Instron, Norwood, MA, USA), following ASTM standard D638. The PLLA/MH composites were cut in dumbbell-shaped specimens (45 × 6 × 2 mm^3^) to measure mechanical properties and the speed was set at 10 mm/min at 25 °C.

### 2.8. Degradation Behavior and pH Change

The PLLA/MH composites were fabricated into a rectangular shape (10 mm × 5 mm, n = 3). Each sample was immersed in a 1.5 mL EP tube of PBS solution (Thermo Fisher Scientific, Waltham, MA, USA) with proteinase K (0.02 mg/mL, Bioneer, Daejeon, Korea) at pH 7.4 at 37 °C. The pH of the PLLA/MH composites was investigated daily at identical times by using a digital pH-meter (Orion Star A211 pH Benchtop Meter, Waltham, MA, USA). The solution in the PLLA/MH composites was removed and dried for 24 h under vacuum conditions then the remaining mass was measured at every time point. The remaining weight was evaluated with the equation below, where $W_{\mathrm{AD}}$ refers to the initial weight of the PLLA/MH composites and $W_{\mathrm{BD}\;}$ refers to the weight of the PLLA/MH composites after decomposition.

(1) $$\mathrm{Weight}\;\mathrm{loss}\;\left(\%\right)=\frac{W_{\mathrm{AD}}}{W_{\mathrm{BD}\;}}\times 100$$

### 2.9. Protein Adsorption

Standard evaluation of protein adsorption on composites was conducted with human plasma fibrinogen. The PLLA/MH composites were placed under PBS solution at 37 °C for 30 min. Fibrinogen solution (0.2 mg/mL) was used to treat the PLLA/MH composites at 37 °C for 1 h. After removing the solution, the composites were rinsed 3 times using distilled water. SDS (5%) was added and incubated overnight at 37 °C. Each well was treated with a micro-bicinchoninic acid assay (BCA, Thermo Fisher Scientific, Waltham, MA, USA) solution for 1 h. Measurement of the absorbance was performed at 562 nm.

### 2.10. Platelet Adhesion

To determine the platelet adhesion of the PLLA/MH composites, specimens with an area of 10 × 10 mm^2^ were fabricated. The specimens were instantly incubated in the platelet solution of 5 × 104 plts/ul at 37 °C. The specimens were rinsed 3 times with PBS solution. Adhered platelets on specimens were lysed by 2% (v/v) Triton X-100 (0.5 mL) for 15 min at 37 °C. Then, the lactate dehydrogenase (LDH) assay was demonstrated according to the manufacturer’s manual (MK401, Takara, Kusatsu, Japan).

The adhered platelets on the specimens were immobilized by glutaraldehyde solution (2.5%) for 1 h and dehydrated sequentially with ethanol aqueous solutions of 50, 60, 70, 80, 90, and 100%. Then, all specimens were coated successively with ethanol: hexamethyldisilane solutions (2:1, 1:1, and 1:2) and then fully dried overnight.

### 2.11. Cell Viability and Inflammation Response Analysis

HCAECs were thawed with 1 × 10^6^ cells and grown in a T75 tissue culture flask with EGM-2 MV. HCAECs were cultured in a humidified incubator at 37 °C with 5% CO_2_. The prepared films (10 × 10 mm^2^) were placed in 24-well plates, sterilized, and hydrated with 70% ethanol for 10 min. HCAECs of 2 × 10^4^ cells were seeded on each composite. After 24 h, cell viability was evaluated using CCK-8. The process was conducted according to the provided protocol. HCAECs were seeded into a 24-well plate at a density of 1 × 10^4^ cells/well, and the cells were stimulated with 12 mM L-Lac, followed by being treated with MH, MH-ODLLA, MH-OLCL7, and MH-OLCL15 (1.1 mg). After 24 h, inflammatory response and cell viability were evaluated using CCK-8 and an ELISA kit (IL-6 and IL-8), respectively. The procedure was conducted according to the provided protocol.

### 2.12. Statistical Analysis

All statistical analyses were conducted using GraphPad Prism 7 (GraphPad Software, Inc., San Diego, CA, USA). One-way analysis of variance (ANOVA) with a Tukey–Kramer multiple comparison test was conducted to contrast the samples [25]. The results were not significant (ns) when *p* > 0.05 and statistically significant when * *p* < 0.05, ** *p* < 0.01, *** *p* < 0.001, and # *p* < 0.0001.

## 3. Results and Discussion

### 3.1. Preparation and Characterization of Surface-Modified MH Nanoparticles

The surface-modified MH nanoparticles were prepared with diverse oligomers and molecular weights. ODLLA, OLCL7, and OLCL15, which were oligomers for surface modification, were synthesized by ring-opening polymerization. ODLLA, OLCL7, and OLCL15 had weight average molecular weight (Mw) of 11,675, 7020, and 15,874, respectively. Characterized by ^1^H NMR, the mole ratios of lactide to caprolactone in OLCL7 and OLCL15 were 62:38 and 63:37, respectively (Table 1).

**Figure 2:**
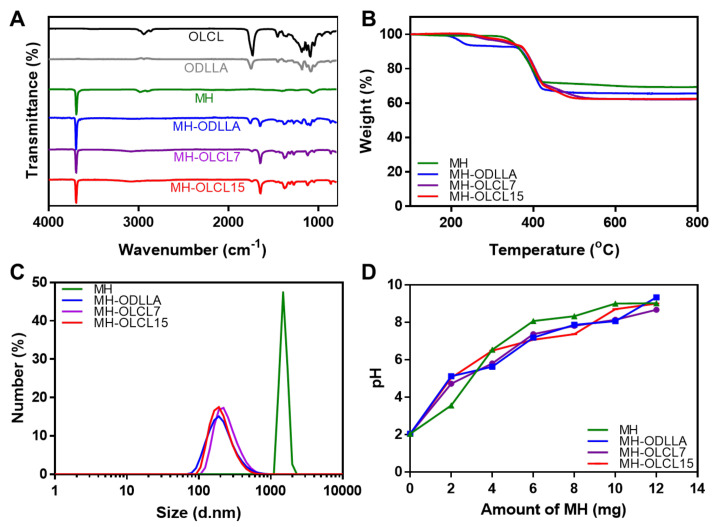
Surface-modified MH characterization. (**A**) ATR-FTIR spectra, (**B**) TGA thermograms, (**C**) size distributions, and (**D**) pH titrations of MH, MH-ODLLA, MH-OLCL7, and MH-OLCL15 nanoparticles.

A chemical analysis of the synthesized MH-ODLLA, MH-OLCL7, and MH-OLCL15 nanoparticles was performed using ATR-FTIR (Figure 2A). In unmodified MH nanoparticles, the peaks appearing at 3699 cm^−1^ are attributed to the −OH stretching vibration. Compared with the unmodified MH nanoparticles, surface-modified MH nanoparticles had the binding vibration peaks (−CH) at 1490–1760 cm^−1^ and the stretching vibration peaks of the carbonyl (−C=O) of ester groups in ODLLA and OLCL. This result suggests that ODLLA and OLCL were successfully modified onto the surface of MH nanoparticles. TGA determined the grafting amounts of oligomers in the MH-ODLLA, MH-OLCL7, and MH-OLCL15 nanoparticles (Figure 2B). Above 600 °C, the residual masses of MH, MH-ODLLA, MH-OLCL7, and MH-OLCL15 were 69%, 65%, 62%, and 62%, respectively. This means that MH-ODLLA, MH-OLCL7, and MH-OLCL15 contained approximately 6%, 10%, and 10% of oligomers, respectively [32,33]. Figure 2C shows the size distribution of MH and surface-modified MH nanoparticles in an organic solvent. The MH, MH-ODLLA, MH-OLCL7, and MH-OLCL15 nanoparticles had average sizes of 1316, 217, 234, and 200 nm, respectively. In organic solvent, MH nanoparticles were aggregated by poor dispersion stability, meanwhile surface-modified MH nanoparticles were maintained due to increased hydrophobicity. In Figure 2D, acid titration analysis was used to evaluate the pH neutralization effect of MH and surface-modified MH nanoparticles. This result shows the acid neutralization capability of pristine MH nanoparticles. It implies that the pH neutralization ability is maintained even after surface modification.

### 3.2. Preparation and Characterization of the PLLA/MH Composites

**Figure 3:**
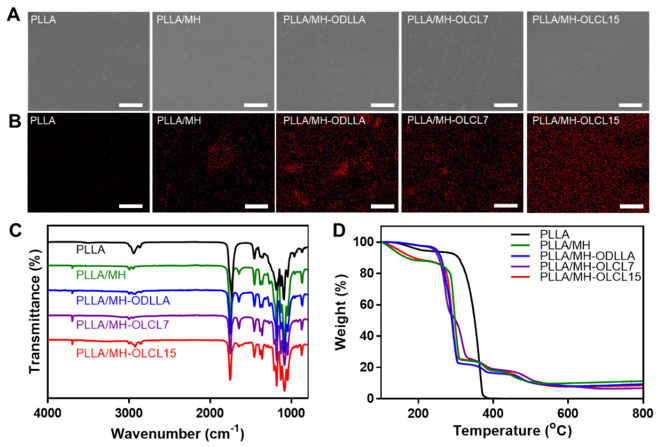
The PLLA/MH composite characterization. Representative (**A**) SEM and (**B**) EDS Mg mapping of the PLLA, PLLA/MH, PLLA/MH-ODLLA, PLLA/MH-OLCL7, and PLLA/MH-OLCL15. Scale bar: 10 μm. (**C**) ATR-FTIR spectra and (**D**) TGA thermograms of each PLLA/MH composite.

The PLLA/MH composites containing surface-modified MH nanoparticles were fabricated using the solvent casting method and hot pressing. When the surface of the PLLA/MH composites was observed through scanning electron microscopy and energy-dispersive X-ray spectroscopy (EDS), all PLLA/MH composites displayed very smooth surfaces (Figure 3A). The distribution of Mg elements in the PLLA/MH composites revealed a difference between MH and surface-modified MH nanoparticles (Figure 3B and Table 2). MH nanoparticles in the PLLA/MH were aggregated, whereas surface-modified MH nanoparticles in the MH-ODLLA, MH-OLCL7, and MH-OLCL15 were evenly dispersed. In particular, MH-OLCL15 showed the highest dispersibility. The water contact angle evaluated the wettability of the PLLA/MH composites. The angles on the PLLA, PLLA/MH, PLLA/MH-ODLLA, PLLA/MH-OLCL7, and PLLA/MH-OLCL15 were 72.31, 48.91, 53.24, 52.19, and 57.90°, respectively. As MH nanoparticles were added, the contact angles decreased, and the surface-modified MH nanoparticles increased the contact angle compared to MH. A chemical analysis of the PLLA/MH composites was performed using ATR-FTIR (Figure 3C). Compared with the PLLA, the PLLA/MH composites had an −OH stretching vibration peak of the MH nanoparticles at 3699 cm^−1^. The proportion of MH nanoparticles in the PLLA/MH composites was analyzed by TGA thermograms. As shown in Figure 3D, All PLLA/MH composites were found to contain approximately 10% of MH nanoparticles.

### 3.3. Mechanical Properties of the PLLA/MH Composites

**Figure 4:**
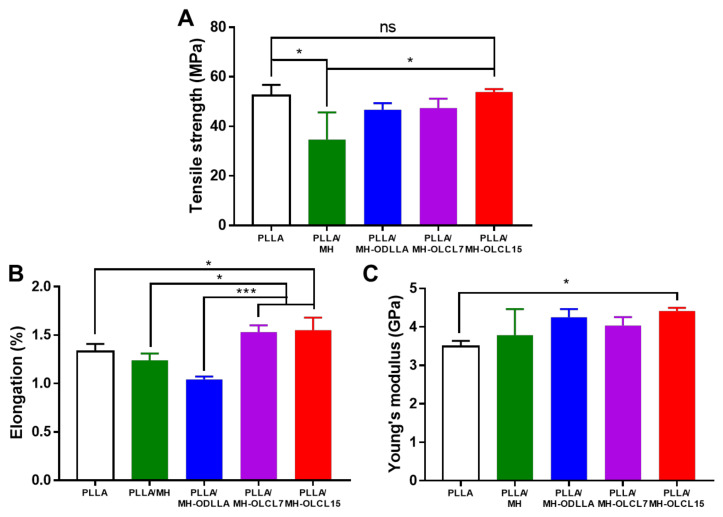
Mechanical properties of the PLLA, PLLA/MH, PLLA/MH-OLCL7, and PLLA/MH-OLCL15. (**A**) tensile strength, (**B**) elongation, and (**C**) Young’s modulus (* *p* < 0.05, ** *p* < 0.01, and *** *p* < 0.001).

The mechanical properties such as tensile strength, elongation, and Young’s modulus of the PLLA/MH composites were investigated as compared with the PLLA composite as a control. Figure 4A displays that the PLLA/MH had lower tensile strength than the PLLA composite, meanwhile when surface-modified MH nanoparticles were included, its tensile strength was gradually recovered, and the PLLA/MH-OLCL15 had similar tensile strength to the PLLA composite. The elongations of PLLA, PLLA/MH, PLLA/MH-ODLLA, PLLA/MH-OLCL7, and PLLA/MH-OLCL15 were 1.33, 1.24, 1.04, 1.53, and 1.55%, respectively (Figure 4B). Compared to the PLLA composite, the elongations of PLLA/MH-OLCL7 and PLLA/MH-OLCL15 increased, while the elongations of PLLA/MH and PLLA/MH-ODLLA decreased. MH nanoparticles in the PLLA matrix are aggregated due to low stability. This phenomenon disrupts the interaction between PLLA molecules. In the case of the PLLA/MH-ODLLA, ODLLA-grafted MH nanoparticles are stable in the PLLA matrix, but ODLLA has semi-crystalline structure like PLLA and reduced the elongation. However, the PLLA/OLCL7 and PLLA/OLCL15 increased the elongation due to the flexible character of OLCL [14]. Young’s modulus significantly increased in the PLLA/MH-OLCL15. Except for the PLLA/MH-OLCL15, the PLLA/MH composites were similar to the PLLA composite (Figure 4C).

### 3.4. Degradation Behavior of the PLLA/MH Composites

**Figure 5:**
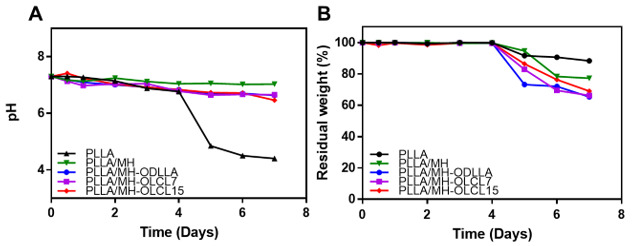
The changes of (**A**) pH and (**B**) residual weight (%) during degradation in proteinase K solution for 7 days at 37 °C.

The degradation of PLLA/MH composites was investigated in the presence of protease K at 37 °C for 7 days in PBS solution [34]. In Figure 5A, the pH of the PLLA composite drastically reduced to pH 4.4 after 7 days of degradation. However, the pH of the PLLA/MH, PLLA/MH-ODLLA, PLLA/MH-OLCL7, and PLLA/MH-OLCL15 dropped slowly for 7 days to reach pH 7.03, 6.66, 6.46, and 6.63, respectively, due to the neutralization ability of MH and surface-modified MH nanoparticles. To overcome the pH decrease of the degradation processing, significant research was performed using inorganic compounds such as calcium carbonate and sodium bicarbonate and they were incorporated into the PLLA to determine their effect on the degradation [35,36]. Especially, MH nanoparticles can neutralize the acidic condition because the dehydrated magnesium ions can bind with part of the anionic compounds [36]. The acidic by-products of PLLA composites were appropriately neutralized by MH nanoparticles, and also the increased formation of degradation products was inhibited by binding of MH to the PLLA composites.

The PLLA/MH, PLLA/MH-ODLLA, PLLA/MH-OLCL7, and PLLA/MH-OLCL15 demonstrated faster weight loss performance than the PLLA composite, which can accelerate the slow degradation of PLLA (Figure 5B). The weight of PLLA and PLLA/MH composites decreased after 4 days. It is known that PLLA maintains mechanical properties for 6 months and accelerates mass loss from 12 months [37]. Therefore, the PLLA/MH composites can maintain mechanical properties for 6 months and biodegrade within 24 months like PLLA. The weight of PLLA only approximately degraded by about 10% for 7 days. Meanwhile, the weight of MH-containing PLLA composites degraded by over 20%. The acidic by-product reacted to the ester linkage of the PLLA composites and then increased the degradation of polymer backbones. In the PLLA composites containing MH nanoparticles, the MH disrupted the accumulation of acidic by-products and precluded intermolecular transesterification and backbiting. These results reveal that the MH influenced the prevention of weight depletion by neutralizing acidic substances.

### 3.5. Blood Compatibility of the PLLA/MH Composites

**Figure 6:**
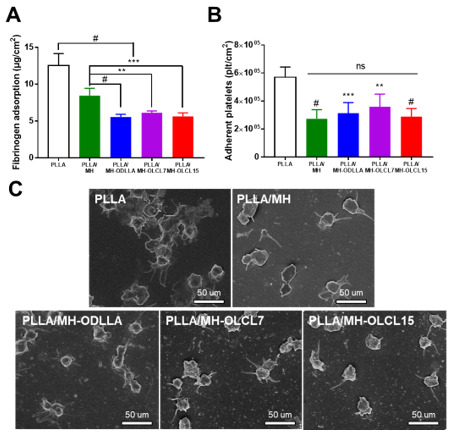
(**A**) Fibrinogen adsorption, (**B**) the number of activated platelets, and (**C**) SEM images of adherent platelets on the surface of PLLA/MH composites. Scale bar: 50 μm (* *p* < 0.05, ** *p* < 0.01, *** *p* < 0.001, and # *p* < 0.0001).

The thrombogenicity of biomaterials is associated with protein adsorption and platelet adhesion. It is well known that fibrinogen causes a cascade of thrombus formation [38]. Accordingly, we tested fibrinogen adsorption and platelet adhesion of the PLLA/MH composites to investigate the blood compatibility. In Figure 6A, it was indicated that the thermal PLLA/MH composites lowered the amount of fibrinogen adsorption as compared with the PLLA composite. Compared with PLLA/MH, the amount of fibrinogen adsorption was further reduced in the composite including surface-modified MH (PLLA/MH-ODLLA, PLLA/MH-OLCL7, and PLLA/MH-OLCL15). The quantitative data using the LDH assay indicated the superiority of anti-platelet adhesion for the PLLA/MH composites as compared with the PLLA composite (Figure 6B). There are four morphologies for the adhered platelets that could govern the activity: (1) round, (2) dendritic, (3) intermediate, and (4) fully spread [39,40,41]. In addition, the activated platelets release potent platelet agonists to aggregate more platelets and finally accelerate the formation of life-threatening thrombi [42]. Figure 6C represents the SEM images of the adhered platelets on PLLA composite and PLLA/MH composites. The PLLA composite exhibited strong activation of adherent platelets on the surface, while the PLLA/MH composites decreased the number of activated platelets.

### 3.6. In Vitro Cell Viability and Anti-Inflammatory Effect

**Figure 7:**
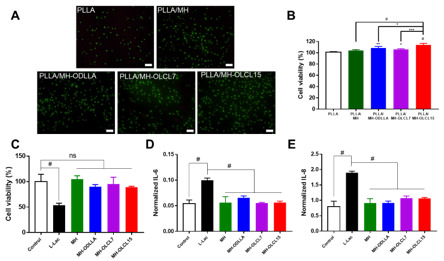
In vitro biocompatibility of the PLLA, PLLA/MH, PLLA/MH-ODLLA, PLLA/MH-OLCL7, and PLLA/MH-OLCL15. (**A**) Live–dead assay images on each PLLA/MH composite at 24 h. Scale bar: 200 μm. (**B**) Cell viability of the HCAECs onto each PLLA/MH composite at 24 h. (**C**) The cell viability, expressions of (**D**) IL-6 and (**E**) IL-8 of HCAECs in L-Lac with MH and surface-modified MH at a concentration of 10 phr (*p < 0.05, **p < 0.01, ***p < 0.001, and #*p* < 0.0001).

In Figure 7A, to investigate cytotoxicity of the PLLA/MH composites, calcein AM and ethidium homodimer 1 (EthD-1) stainings were performed with HCAECs at 24 h. Dead cells were rarely exhibited on the PLLA composite and PLLA/MH composites. However, live cells increased on the PLLA/MH composites compared to the PLLA composite. Among them, PLLA/MH-OLCL15 observed the highest cell growth.

In Figure 7B, the cell viability was quantified using CCK-8 at 24 h. HCAEC growth increased to 104.07, 108.57, 106.03, and 114.01% in the PLLA/MH-ODLLA, PLLA/MH-OLCL7, and PLLA/MH-OLCL15, compared to the PLLA composite. Likewise, for live–dead staining, PLLA/MH-OLCL15 showed the highest cell viability (*p* < 0.0001). Since a relatively hydrophilic surface improves the adhesion of the cells [43], Mg^2+^ released from MH directly affects the function of HCAECs and induces endothelialization [29]. The toxicity and inflammatory response of MH and surface-modified MH nanoparticles were evaluated under degraded acidic by-products of PLLA. In Figure 7C, the viability of HCAECs in the L-Lac group decreased significantly and the cell viability was less than 50%. This suggests that degraded acidic by-products of PLLA are toxic to HCAECs. In contrast, MH, MH-ODLLA, MH-OLCL7, and MH-OLCL15 were maintained in cell viability by the neutralizing effect under degraded acidic by-products of PLLA. The ideal characteristic of biomedical devices is to attenuate proinflammatory cytokines such as IL-6 and IL-8 after implantation [44,45,46,47]. In Figure 7D and 7E, the inflammation response of HCAECs was analyzed by ELISA (IL-6 and IL-8) using conditioned media, and normalized to cell number assessed by the cell viability. It was observed that both IL-6 and IL-8 significantly improved in the L-Lac treated group compared to that in the negative control group. Compared to the L-Lac treated group, the expression of IL-6 and IL-8 remarkably decreased in MH and surface-modified MH nanoparticles. There was no significant difference in the inhibition of expression of inflammatory cytokines between the MH-ODLLA, MH-OLCL7, and MH-OLCL15. This result implies that both the MH and surface-modified MH sufficiently neutralized the acidic decomposition products of PLLA to inhibit the inflammatory response.

## 4. Conclusions

In this study, hydrophobic-oligomer-modified MH nanoparticles were contained in the PLLA matrix to inhibit inflammatory reactions caused by acidic degradation products of PLLA. Hydrophobic oligomers were successfully modified on the MH surface through a nucleophilic addition reaction. The PLLA/MH composites with surface-modified MH nanoparticles showed high mechanical properties and an acid-neutralizing effect. Especially, compared to the PLLA/MH-ODLLA, PLLA/MH-OLCL7 and PLLA/MH-OLCL15, composites increased not only in Young’s modulus and tensile strength but also in elongation. In an in vitro cell experiment, anti-inflammation and low cytotoxicity of MH nanoparticles were maintained after surface modification. In particular, the PLLA/MH-OLCL15 composite exhibited higher biocompatibility than the PLLA composites. The protein adsorption and platelet adhesion confirmed that the MH and surface-modified MH composites decreased fibrinogen adsorption and platelet activation, which indicates their excellent blood compatibility. Although long-term and detailed molecular biological analyses and in vivo toxicity experiments will be required in the future, this study proved that surface-modified MH with oligomers could be a potent additive to improve the biocompatibility of the polymer matrix including biodegradable PLLA for various biomedical applications.

## Figures and Tables

**Table 1:** Characteristics of oligomers.

Oligomer	Feed Ratio (mol.%)		Product Ratio (mol.%)		Mw
	Lactide	Carprolactone	Lactide	Carprolactone	
ODLLA	100	-	100	-	11,675
OLCL7	70	30	62	38	7020
OLCL15	70	30	63	37	15,874

**Table 2:** SEM-EDS analysis and water contact angle on the surface of the PLLA/MH composites.

PLLA/MH Composite	Atomic Composition (%)			Water Contact Angle (°)
	C	O	Mg	
PLLA	63.71	36.29	-	72.31 ± 1.01
PLLA/MH	62.44	35.78	1.78	48.97 ± 2.71
PLLA/MH-ODLLA	63.72	33.97	2.31	53.24 ± 1.36
PLLA/MH-OLCL7	60.27	37.54	2.19	52.19 ± 2.25
PLLA/MH-OLCL15	63.24	34.63	1.71	57.90 ± 1.51

## Data Availability

The data presented in this study are available in this study. Additional information could be available on request from the corresponding author.
